# Encapsulation Mechanism of Oxyresveratrol by β-Cyclodextrin and Hydroxypropyl-β-Cyclodextrin and Computational Analysis

**DOI:** 10.3390/molecules22111801

**Published:** 2017-10-31

**Authors:** Jianfei He, Zong-Ping Zheng, Qin Zhu, Fengxian Guo, Jie Chen

**Affiliations:** 1 State Key Laboratory of Food Science and Technology, Jiangnan University, Wuxi 214122, China; Jenniferhejf@gmail.com; 2 Fujian Province Key Laboratory for the Development of Bioactive Material from Marine Alge, College of Oceanology and Food Science, Quanzhou Normal University, Quanzhou 362000, China; guofx0608@126.com; 3 Key Lab of Medical Plant Genetic Improvement and Quality Control of Zhejiang Province, College of Life and Environmental Sciences, Hangzhou Normal University, Hangzhou 311121, China; zhuqin@hznu.edu.cn

**Keywords:** oxyresveratrol, encapsulation constant, pH, temperature, thermodynamic parameters, molecular docking

## Abstract

In this study, the encapsulation mechanism of oxyresveratrol and β-cyclodextrin (β-CD) and hydroxypropyl-β-cyclodextrin (HP-β-CD) was studied. As this research shows, oxyresveratrol and two cyclodextrins (CDs) were able to form inclusion complexes in a 1:1 stoichiometry. However, the interaction with HP-β-CD was more efficient, showing up as higher encapsulation constant (*K_F_*) (35,864.72 ± 3415.89 M^−1^). The *K_F_* values exhibited a strong dependence on temperature and pH, which decreased as they increased. From the thermodynamic parameters (Δ*H*^0^, Δ*S*^0^, and Δ*G*^0^) of the oxyresveratrol loaded β-CD (oxyresveratrol-β-CD) and HP-β-CD (oxyresveratrol-HP-β-CD), it could be seen that the complexation process was spontaneous and exothermic, and the main driving forces between oxyrsveratrol and CDs were hydrogen bonding and van der waals force. Besides, molecular docking combined with ^1^H-NMR were used to explain the most possible mode of interactions between oxyresveratrol and CDs.

## 1. Introduction

A supramolecule is an advanced structure with certain complexity and organization. Cyclodextrins (CDs) are most commonly used in supramolecular chemistry due to their special spatial structures [1,2]. Today, especially in the pharmaceutical field, cyclodextrin (CD) has the ability to form inclusion complexes with a variety of guest molecules to help improving their physicochemical properties without modification of original structure and thus have attracted wide attention [3].

CD inclusion is characterized by the absence of covalent bonds and a definite stoichiometric ratio of guest and CD, which dissolves and decomposes rapidly in aqueous solution and reach the dynamic equilibrium between the free component and the complex in a few seconds [4]. Several types of forces are involve in the inclusion complex formation, including electrostatic interaction, van der Waals interaction, hydrophobic interaction, hydrogen bonding, reduction of conformational strain, exclusion of cavity-bound high-energy water, and charge-transfer interaction. These forces directly affect the stability of the inclusion complex, while their relative contributions depend on many factors such as the guest and CD type [5,6] and the temperature and pH of the system [7,8].

The inclusion constant (*K_F_*) is an important index used to measure the binding strength of the complex and the physical and chemical properties of the guest molecule. The determination of the value of inclusion constant is one of the critical step to judge whether cyclodextrin can be applied to pharmaceutical preparations. Therefore, it usually acts as an initial point for drug-cyclodextrin application research. In general, if the *K_F_* value is too large, the balance will move into the formation of complex [9]. *K_F_* values combine with the change of the values of thermodynamic parameters (Δ*H*^0^, Δ*S*^0^, and Δ*G*^0^) are commonly used to investigate the main driving forces between cyclodextrin and specific guest molecules.

Researchers recently studied the inclusion complexes of resveratrol with both natural and modified CDs [10,11]. The results showed that CD complexes greatly increased the water solubility and antioxidant activity of the drug, particularly when it was complexed with HP-β-CD [12,13,14]. Oxyresveratrol is a kind of natural polyhydroxylated stilbene with poor aqueous solubility and sensitivity to external environment, highly similar to resveratrol [15]. The results of resveratrol indicate that oxyresveratrol may also have the possibility to be incorporated with CD. 

**Figure 1:**
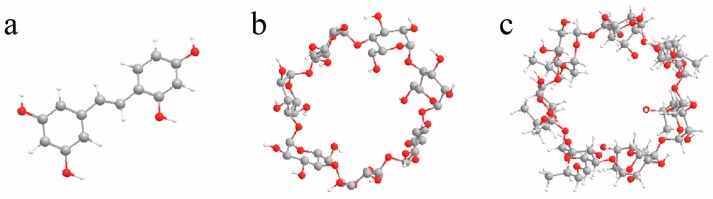
3D molecule structures of oxyresveratrol (**a**); β-CD (**b**); HP-β-CD (**c**).

The purpose of this work was to clarify the encapsulation mechanism of oxyresveratrol (Figure 1a) by natural and modified CDs (β-CD and HP-β-CD) (Figure 1b,c) and evaluated the effects of temperature and pH values on the combination ability of oxyresveratrol and CDs. Molecular docking and ^1^H-NMR were used to further study the types of interactions between oxyresveratrol and CDs.

## 2. Results and Discussion

### 2.1. Stoichiometry Determination

**Figure 2:**
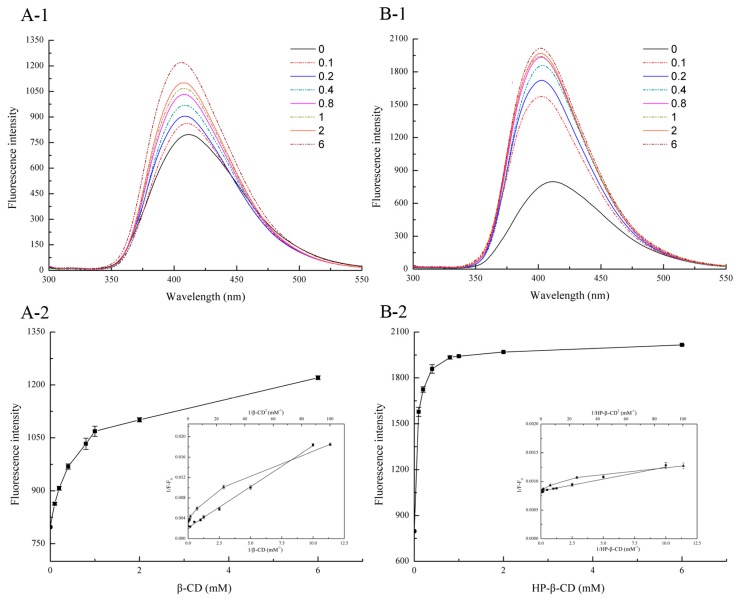
Differential fluorescence emission spectrums of oxyresveratrol/β-CD (**A-1**) and oxyresveratrol/HP-β-CD (**B-1**) inclusion complexes against oxyresveratrol at the same concentration (100 μM). 0 → 6 mean the concentration of cyclodextrin at 0, 0.1 mM, 0.2 mM, 0.4 mM, 0.8 mM, 1.0 mM, 2.0 mM and 6.0 mM. Dependence of emission fluresence intensities of oxyresveratrol on varies β-CD (**A-2**) and HP-β-CD (**B-2**) concentrations. Inset: double reciprocal plot of oxyresveratrol complexed to cyclodextrins for determining the stoichiometry of oxyresveratrol/CD conplexes: 1/(F_∞_ − F_0_) versus 1/[CD] (assumption of 1:1 complex) (filled squares); 1/(F_∞_ − F_0_) versus 1/[CD]^2^ (assumption of 1:2 complex) (filled triangles).

The effect of CD on the fluorescence intensity of the system was revealed in Figure 1. From Figure 1, it can be found that the fluorescence intensity of oxyresveratrol solution increased gradually with the increase amount of CD, accompanied by the 5 nm blue shift of maximum emission wavelength from 411.5 nm to 406.5 and 401.5 nm for β-CD as well as HP-β-CD, respectively (Figure 2(A-1,B-1)). This phenomenon can be interpreted as follows: After forming an inclusion complex with the guest molecule, the appropriate hydrophobic microenvironment in the CD cavity helps to reduce the interaction between the guest molecule and the aqueous solution or quencher [16]. Comparing the fluorescence enhancement of two kinds of CDs at the same concentration (Figure 2(A-2,B-2)), it was easy to find that HP-β-CD was able to form complex with oxyresveratrol at lower concentration than β-CD, indicating its stronger inclusion capacity. However, under high concentration, the fluorescence intensity varied little with the concentration of CD, almost constant or fluctuated at a certain value, which may be caused by the certain fluorescence quenching effect on the phosphor [17]. As shown in Figure 2(A-2,B-2) inset (filled squares), the double-reciprocal plot of 1/(F_∞_ − F_0_) versus 1/[CD] gave a straight line with a correlation coefficient of 0.99, indicating that the stoichiometry of the oxyresveratrol-CD interaction was 1:1. In addition, a non-linear relationship was obtained from the plot of 1/(F_∞_ − F_0_) versus 1/[CD]^2^ (filled triangles), indicating that the stoichiometric ratio of the inclusion compound was not 1:2 [18].

### 2.2. Temperature Effects

**Figure 3:**
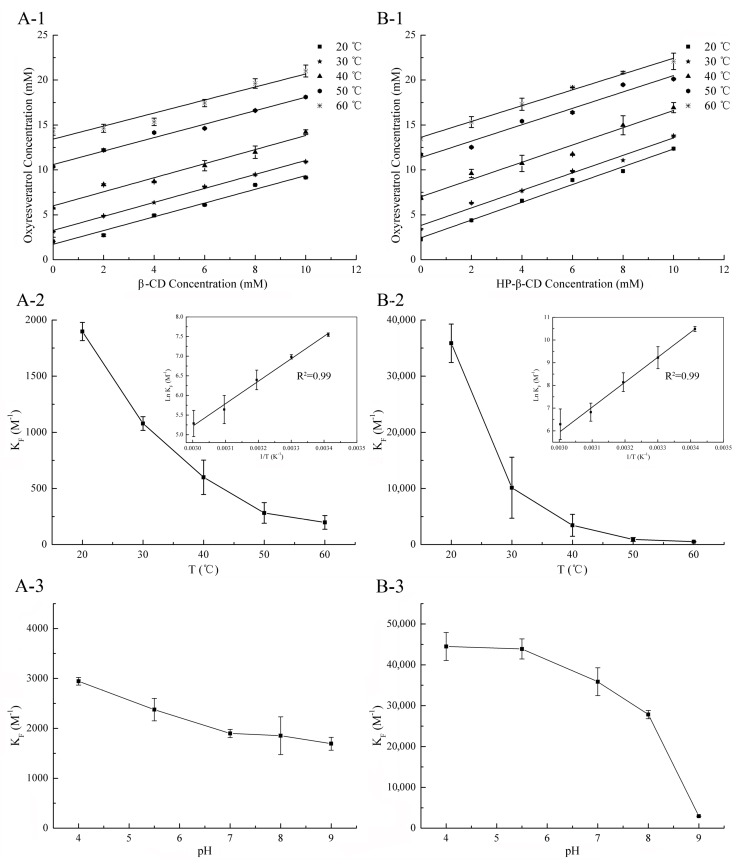
Oxyresveratrol/β-CD (**A-1**) and oxyresveratrol/HP-β-CD (**B-1**) phase solubility diagrams at different temperatures. Effect of temperatures on the complexation constant (*K_F_*) of oxyresveratrol-β-CD inclusion complex (**A-2**) and oxyresveratrol-HP-β-CD inclusion complex in H_2_O (**B-2**). Inset: the Van’t Hoff plot (ln *K_F_* versus 1/T) for oxyresveratrol-β-CD (**A-2**) and oxyresveratrol-HP-β-CD (**B-2**) inclusion complexes. Effect of pH on the *K_F_* of oxyresveratrol-β-CD inclusion complex (**A-3**) and oxyresveratrol-HP-β-CD inclusion complex (**B-3**). (*n* = 3, if no error bars are displayed, errors were smaller than symbols).

Many previous studies have shown that temperature has a significant effect on the inclusion reaction [19,20,21]. In order to control the temperature and reduce the influence of temperature change in the experiment process, the phase solubility method was used to calculate the inclusion constant. As shown in Figure 3, the solubility of oxyresveratrol increased linearly (*R*^2^ ≥ 0.99) with the increasing concentration of β-CD and HP-β-CD, which can be defined as classic A_L_-type phase-solubility diagrams, meaning that oxyresveratrol and these two CDs were able to form inclusion complexes in a 1:1 stoichiometry [22,23], consistent with the results obtained by fluorescence testing. The stability constant values of oxyresveratrol-β-CD and oxyresveratrol-HP-β-CD at 20 °C were found to be 35,864.72 ± 3415.89 M^−1^ and 1897.54 ± 81.14 M^−1^, respectively. The higher *K_F_* value of HP-β-CD demonstrated that it complexed better with guest oxyresveratrol molecule than that of β-CD. Comparing the β-CD, the addition of hydroxypropyl substituents in HP-β-CD helped to deeper the cavity and increase the hydrophobicity of molecule [24], which provided a better inclusion environment and result in its stronger binding capacity [25]. 

On the other hand, the findings obtained from phase solubility diagrams at different temperatures (Figure 3(A-1,B-1)) confirmed that the oxyresveratrol solubility in the presence of β-CD and HP-β-CD increased as the temperature increment, which might be attributed to the release of bound water molecules inside the CD cavity at higher temperature [26]. The *K_F_* values of both two CDs decreased with temperature rising, from 1897.54 ± 81.14 M^−1^ to 197.74 ± 61.29 M^−1^ and 35,864.72 ± 3415.89 M^−1^ to 507.61 ± 43.78 M^−1^ for oxyresveratrol-β-CD and oxyresveratrol-HP-β-CD in 20 °C and 60 °C, respectively (Figure 3(A-2,B-2)), different from the previous report [27]. The results indicated that the tendency to form inclusion was reduced, which might be attributed to the fact that hydrogen bonds are weakened by heating [19,20,21].

### 2.3. Thermodynamics Analysis

Thermodynamic parameters including Δ*H*^0^, Δ*S*^0^, and Δ*G*^0^ were listed in Table 1, which vary according to the type of host-guest interaction, among them Δ*S*^0^ and Δ*G*^0^ were calculated by the formula above (6 and 7), while the values of Δ*H*^0^ can be directly obtained from inset plot in Figure 3. As shown in Figure 3(A-2,B-2), the plot of ln *K_F_* versus 1/T was linear, with a correlation coefficient higher than 0.99. On the other hand, the values of Δ*G*^0^, Δ*H*^0^, and Δ*S*^0^ were negative at all five temperatures. From the thermodynamic standpoint, the complexation processes between oxyresveratrol and CDs were spontaneous and exothermic, and the translational and rotational degree of freedom of the complexed guest molecules was reduced when complexation occurred, leading to a more ordered system [28]. According to the second law of thermodynamics, negative Δ*S*^0^ value was not conducive to the reaction, but its adverse effect can be offset by the Δ*H*^0^, which was much larger and acted as the main driving force in the inclusion process between oxyresveratrol and these two CDs [29]. The change of the thermal function was less than that of the general chemical reaction, indicating that there was no covalent bond formation, which may be the result of the intermolecular hydrogen bond and van der waals force [30]. Moreover, the thermodynamic parameters of Oxy and two CDs are quite different. Compared with β-CD, the absolute value of Δ*H*^0^, Δ*G*^0^, and Δ*S*^0^ in the reaction of Oxy with HP-β-CD was higher than that of β-CD. The results indicated a more intense exothermic reaction, higher ordering degree of drug molecule and tendency to form inclusion complex in the inclusion process with HP-β-CD, which possibly due to the stronger hydrophobicity of the HP-β-CD cavity.

### 2.4. pH Effects

As reported in many studies, *K_F_* values have a strong pH dependency in both natural and modified CDs [4,19]. As shown in Figure 3(A-3), the *K_F_* value of oxyresveratrol-β-CD decreased from 2944.42 ± 75.69 M^−1^ (pH 4.00) to 1692.97 ± 129.91 M^−1^ (pH 9.01), whereas oxyresveratrol-HP-β-CD *K_F_* value decreased sharply from 44495.78 ± 3417.78 M^−1^ (pH 4.00) to 2937.36 ± 3.67 M^−1^ (pH 9.01). The *K_F_* observed in Figure 3(B-3) sharply decreased in region where deprotonation of the hydroxyl groups of oxyresveratrol, suggesting that the strength of hydrogen bonds played an important role in stabilizing of inclusion complexes. Meanwhile, the *K_F_* value decreased with the increasing of pH value during the formation of inclusion complexes between oxyresveratrol and CDs indicated that alkaline condition was not suitable for the preparation and usage of inclusion complex. On the other hand, the higher *K_F_* value of oxyresveratrol-HP-β-CD suggested that complexes formed between HP-β-CD and the protonated form of oxyresveratrol were more stable than those formed with β-CD. 

### 2.5. ^1^H-NMR Analysis

^1^H-NMR spectrum is one of the most direct evidence for determining the structure of the inclusion complex according the chemical shift displacement of host and guest molecules [31]. After the formation of inclusion complex, the chemical shifts variations (Δδ, Δδ = δ_complex_ − δ_free_) of host and guest molecules, which is caused by the ring current effects generated by circulating π electrons of the aromatic guest [32,33], can be observed. It is well known that the chemical shifts variation of H-3 and H-5 protons located in the inner surface of the CD cavity are more easily observed than that of the protons H-1, H-2, H-4, and methylene H-6 located at the outer surface. Moreover, H-3 and H-5 are positioned at different locations of the internal cavity, while H-3 protons are at the wide rim and H-5 protons are close to the narrow side [34]. Therefore, the observed magnitude of their displacements provides some binding mode information of CD inclusion complexes [26,35].

**Figure 4:**
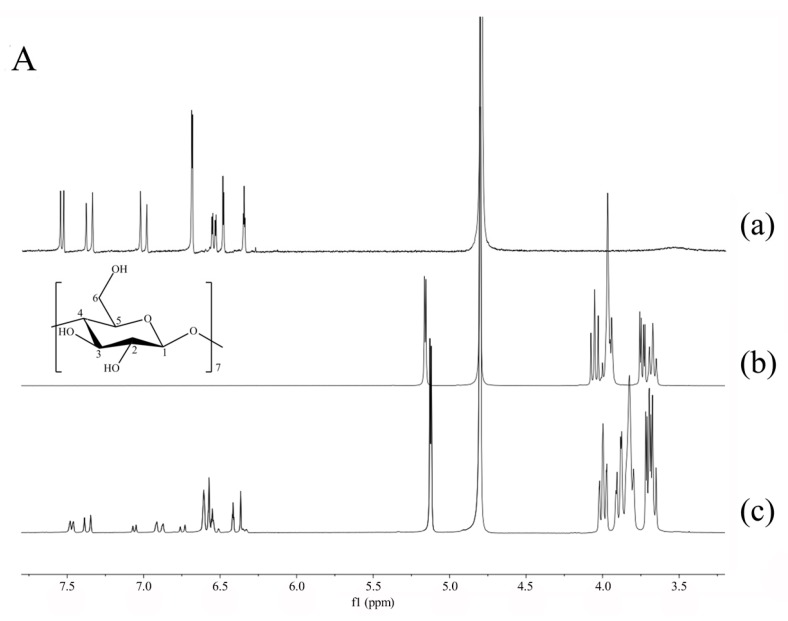

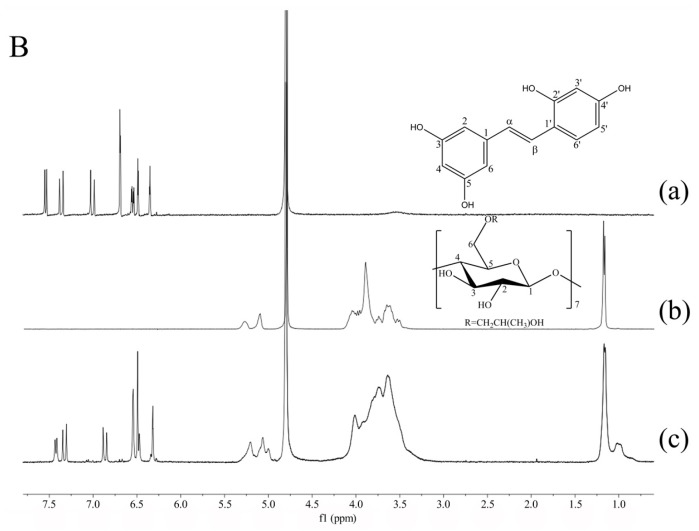
^1^H-NMR spectra of oxyresveratrol (**a**); β-CD (**b**); oxyresveratrol-β-CD inclusion complex (**c**) in D_2_O (**A**); ^1^H-NMR spectra of oxyresveratrol (**a**); HP-β-CD (**b**); oxyresveratrol-HP-β-CD inclusion complex (**c**) in D_2_O (**B**).

To get more insights into the inclusion mode of oxyresveratrol-β-CD, the ^1^H-NMR signals of oxyresveratrol, β-CD and oxyresveratrol-β-CD inclusion complex in D_2_O were compared. As presented in Figure 4(A-a,b) it was obvious that almost all protons of oxyresveratrol and β-CD proved to be affected by the inclusion process, expressed as a positive and negative chemical shift on the NMR spectrum. The specific chemical shifts of oxyresveratrol and β-CD protons before or after complexing were compared in Table 2. As described in Table 2, the upfield shifts (Δδ) could be observed from the H-3 (−0.055 ppm) and H-5 (−0.045 ppm) protons of β-CD. In general, Δδ H-3 > Δδ H-5 or Δδ H-3 < Δδ H-5 indicated of partial or total inclusion of the guest inside the CD cavity [36,37]. It can be speculated that the guest molecule entered the CD cavity from the wide side and was directed toward the narrow side [27].

It can be observed from Figure 4(A-a) and Table 2 that the protons of free oxyresveratrol appeared at δ between 6.25 and 7.75 ppm, outside the protons region of β-CD. The intensity of the signals from oxyresveratrol were low due to the small percentage in the complex (Figure 4(A-c)), while the signals of the guest were still clearly differentiable. As a consequence of the de-shielding and shielding effect, the chemical shifts (Δδ) of oxyresveratrol aromatic ring protons at H-2, H-4, H-6, H-3’, H-5’, and H-6’ were of −0.083, 0.140, −0.083, 0.065, 0.001, and −0.071 ppm after the formation of complex, indicating that both two aromatic rings of oxyresveratrol was inserted into the cavity of β-CD and oriented to the narrow side [38]. Thus, it can be proposed that oxyresveratrol could form inclusion complex with β-CD, but the molecule did not completely pass through the cavity.

The variations of chemical shifts of HP-β-CD and oxyresveratrol-HP-β-CD protons were also depicted in Figure 4B and Table 2. Based on the result obtained, including H-3 and H-5 protons showed Δδ values of −0.021 ppm and −0.004 ppm, as well as the characterized protons of aromatic ring possessed visible chemical shifts. The inclusion mode of oxyresveratrol/HP-β-CD was similar to that of β-CD. The oxyresveratrol-HP-β-CD inclusion complex was formed by penetrating aromatic rings of guest molecule into the cavity from the wide rim of HP-β-CD cavity partly. In addition, the protons of oxyresveratrol in HP-β-CD complex presented higher degrees of Δδ changes than β-CD, which might be caused by the stronger H-bond interaction between them [35].

### 2.6. Molecular Docking Studies

**Figure 5:**
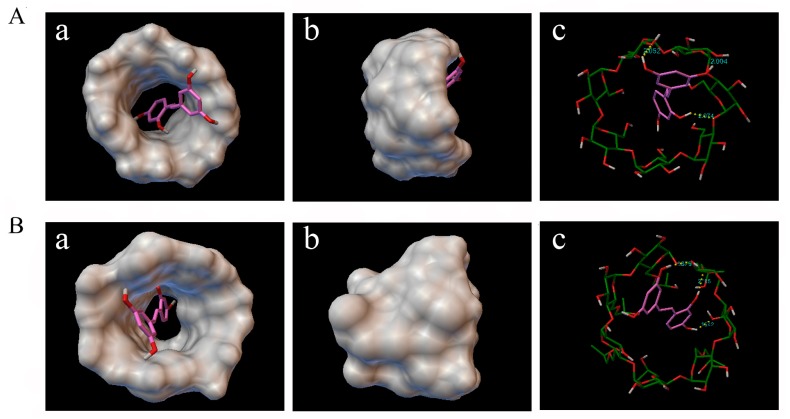
Docking calculation results and proposed favorable mode of the inclusion complex (**a**) top view from the wide rim of oxyresveratrol-β-CD; (**b**) side view of oxyresveratrol-β-CD; and (**c**) stick molecular representation of oxyresveratrol-β-CD (**A**); (**a**) top view from the wide rim of oxyresveratrol-HP-β-CD; (**b**) side view of oxyresveratrol-HP-β-CD; and (**c**) stick molecular representation of oxyresveratrol-HP-β-CD (**B**). In yellow, hydrogen bonds.

Molecular docking was carried out to get further insight about how oxyresveratrol interacts with β-CD and HP-β-CD. According to all of the docking results, there was no case that the oxyresveratrol molecule was away from the CD cavity, indicating oxyresveratrol was able to form a stable inclusion compound with β-CD and HP-β-CD theoretically. The cluster analysis of the docking results showed that the docking experiment was in good convergence since the best docked cluster included 356 and 392 kinds of docking configurations for β-CD and HP-β-CD, respectively, in 500 times docking runs. And the minimum energy conformation of the best docked cluster (Figure 5) was always used to analyze the docking chemical structure. As shown in Figure 5A, the oxyresveratrol molecule was not encapsulated into the cavity of CD completely; one of its hydrophobic benzene ring was deeply buried into the β-CD cavity and was close to the narrow rim of β-CD, while another was located at the wide rim of the CD, which was consistent with the ^1^H-NMR results. Furthermore, molecular docking calculations predicted interatomic distances of three H-bonds between oxyresveratrol and β-CD (Figure 5(A-c)) to be 2.052, 2.074 and 2.004 Å, respectively. These H-bonds played a key role in stabilizing the inclusion complex. In addition, as depicted in Figure 5B, similar to the inclusion of oxyresveratrol-β-CD, oxyresveratrol could form inclusion complexes with HP-β-CD, which was stabilized by three H-bonds, and their interatomic distances were found to be 2.105, 1.942, and 1.891 Å, respectively. In general, the shorter distance between atoms would cause more ineffective van der Waals and more stable hydrogen bonds, which could explain why the binding constants of HP-β-CD were larger than that of β-CD and was more sensitive to temperature changes. The findings were in good agreement with the results of other studies [23,39].

We also calculated the interaction energies of these complexes (Table 3). All complexes had the same torsional energy. The substitution caused different intermolecular energies, internal energies and unbound energy. As a result, significant differences in binding energies formed. The negative binding energy changed upon complexation clearly demonstrated that β-CD and HP-β-CD can form stable complexes with oxyresveratrol, which was observed in the experiments. The binding energy of oxyresveratrol-HP-β-CD complex was lower than that of the oxyresveratrol-β-CD inclusion complex, which suggested that the substitution of 2-hydroxypropyl group could improve the inclusion capacity of β-CD, as determined by the fluorescence spectroscopy and thermodynamics results.

## 3. Materials and Methods

### 3.1. Materials

HP-β-CD and β-CD were purchased from Jiangsu Fengyuan Biotechnology Co., Ltd. (Suqian, China). Oxyresveratrol was purchased from Hangzhou Great Forest Biomedical Ltd. (Hangzhou, China). Sodium dihydrogen phosphate and disodium phosphate were purchased from Sinopharm Chemical Reagent Co., Ltd. (Shanghai, China). HPLC grade methanol were purchased from J&K Scientific (Beijing, China). HPLC grade formic acid was purchased from Shanghai Aladdin Chemical Reagent Co., Ltd. (Shanghai, China).

### 3.2. Methods

#### 3.2.1. Fluorescence Spectroscopy

A Hitachi F-2700 spectrofluorimeter (Shimadzu, Japan) equipped with a xenon lamp source and a 1.0 cm quartz cell was used to obtain fluorescence spectra. The emission wavelength of oxyresveratrol was 288 nm, while the excitation spectra were recorded from 300 to 550 nm at room temperature (25 °C). The emission bandwidths were set as 10 nm. The complex solutions were prepared to a final volume of 5 mL by adding 100 μM oxyresveratrol to CD solution of different concentrations, which varied from 0 to 6 mM under stirring. All reagents were dissolved in water.

#### 3.2.2. Study of the Stoichiometry

For the CD inclusion compound having a stoichiometric ratio of 1:1 and 1:2, there are balances as follow:

Oxyresveratrol + CD → Oxyresveratrol-CD(1)

Oxyresveratrol + 2CD → Oxyresveratrol-2CD(2)

According to these balances, two mathematical models were proposed to determine the stoichiometry of the oxyresveratrol-CD interaction, Equations (3) and (4) represent models 1:1 and 1:2, respectively.

1/(*F* − *F*_0_) = 1/((*F*_∞_ − *F*_0_)·*K_F_* [CD]) + 1/(*F*_∞_ − *F*_0_) (3)

1/(*F* − *F*_0_) = 1/((*F*_∞_ − *F*_0_)·*K_F_* [CD]^2^) + 1/(*F*_∞_ − *F*_0_) (4)

where [CD] is the concentration of CD, *K_F_* is the encapsulation constant, *F*_∞_ is the fluorescence intensity when the total oxyresveratrol has been complexed with CD, *F*_0_ is the fluorescence intensity in the absence of CD, and *F* is the observed fluorescence intensity.

If the stoichiometry of oxyresveratrol and CD is 1:1, the representation of 1*/*(*F*_∞_ *− F*_0_) versus 1/[CD] should give a linear plot. Otherwise, if order inclusion complexes (1:2) are higher, the double-reciprocal plot of 1/(*F*_∞_ *− F*_0_) versus 1/[CD]^2^ will be linear.

#### 3.2.3. HPLC-DAD Analysis of Oxyresveratrol

HPLC analyses were performed using a Waters 2707 HPLC. The separation was performed in a reverse-phase Grace Smart (4.6 μm, 2.1 × 250 mm, Ryss Tech Ltd., Shanghai, China) with a mobile phase of 0.1% formic acid (solvent A) and methanol (solvent B). Flow rate was 1.0 mL/min, and the effluent was monitored at 325 nm with a Waters 2487 dual-wavelength absorbance detector. Three injections were performed for each sample. The calibration curve of oxyresveratrol in the concentration range from 3.125 to 200 μg/mL was linear with a correlation coefficient of 0.9999.

#### 3.2.4. Phase Solubility Study and Encapsulation Constant Determination

Phase solubility studies were performed in water at 20, 30, 40, 50 and 60 ± 0.5 °C, respectively, using a hot-plates (C-MAG HS 7, IKA, Wilmington, NC, USA) to control the temperature, according to the method previous reported [14]. In brief, excess amount of oxyresveratrol was added to 5 mL β-CD and HP-β-CD solutions in distilled water with concentration among 0 to 10 mM. The mixtures were continuous stirred (GENIUS 3 vortex and RW 20D magnetic stirrer, IKA laboratory technology, Germany) for 48 h until solubility equilibria was reached. The mixed solution was then filtered through 0.2 μm microporous membrane filters. The obtained filtrate was diluted appropriately with deionized water and the oxyresveratrol concentration in aqueous solution was analyzed by HPLC as reported above.

According to the phase solubility diagram, the encapsulation constant *K_F_* of the complex having a stoichiometric ratio of 1:1 can be determined by the Equation (5):

*K_F_* = slope/S_0_ (1 − slope) (5)

where S_0_ is the solubility of oxyresveratrol in aqueous solution without the existence of CD. 

#### 3.2.5. Determination of Thermodynamic Parameters

The thermodynamic parameters Δ*H*^0^, Δ*S*^0^, and Δ*G*^0^ can be estimated from the Gibbs and Van’t Hoff equation:

Δ*G*^0^ = −R*T*·Ln *K_F_* (6)

Ln *K_F_* = −Δ*H*^0^/R*T* + Δ*S*^0^/R (7)

where *T* is the stirring temperature, R is the universal gas constant approximate 8.314 J/(mol·K), Δ*G*^0^ is Gibbs free energy change during the inclusion process, Δ*H*^0^ and Δ*S*^0^ are standard enthalpy, and entropy changes of complex formation in the mobile phase. For a linear plot of ln *K_F_* versus 1/T, −Δ*H*^0^/R and Δ*S*^0^/R play a role as slope and intercept, respectively.

#### 3.2.6. pH Effects

The medium in acidic or alkaline is usually used in food industry, so pH is an important factor affecting the inclusion process [7], and it is necessary to clear the effect of protonation state on inclusion ability. The *K_F_* values for the oxyresveratrol-β-CD and oxyresveratrol-HP-β-CD complexes were determined in the pH range 4.00–9.01 in this study. Different buffer solutions were used to control the pH: pH (3–5.5) sodium dihydrogen phosphate, pH (5.5–8.0) the mixture of sodium dihydrogen phosphate and disodium phosphate, pH (8.0–12.0) disodium phosphate.

#### 3.2.7. Nuclear Magnetic Resonance (NMR) Spectroscopy

Proton NMR spectra of oxyresveratrol, CDs, and inclusion compounds were taken on an AVANCE III 400 MHz Digital NMR spectrometer (Brucker Co., Billerica, MA, USA). The chemical shifts (δ) were expressed in ppm. Samples of about 5 mg were dissolved in 0.5 mL D_2_O.

#### 3.2.8. Molecular Docking

AutoDock 4.2 (The Scripps Research Institute, La Jolla, CA, USA) was utilized in this study to predict the most likely optimal configuration of the inclusion complex oxyresveratrol-β-CD and oxyresveratrol-HP-β-CD. The structure of β-CD was extracted from a crystal structure from the Protein Data Bank (PDB ID: 1z0n) while HP-β-CD was built by adding hydroxypropyl to β-CD. The three-dimensional (3D) structure of CDs were generated using ChemBio3D Ultra 12.0 (CambridgeSoft, Cambridge, MA, USA) program and then optimized with semi empirical (PM3) energy minimization. The molecular structure of oxyresveratrol was drawn in ChemBioDraw Ultra 12.0 (CambridgeSoft) and treated by the DFT/B3LYP method in ChemBio3D Ultra 12.0. The non-polar hydrogen atoms were emerged, and the gasteiger charges were added for all atoms of optimized molecules using AutoDockTools. AutoGrid 4.2 was used to calculate the grid maps before docking. The resolution of grid was 40 × 40 × 40 points, with a grid spacing of 0.375 Å. The three substrates were separately docked into this grid with the genetic algorithm (GA) as implemented in AutoDock 4.2. Default parameters were used, except for the number of generations, energy evaluations, and docking runs, which were set to 150, 2,500,000 and 500, respectively, and the resulting docking mode was visualized.

#### 3.2.9. Statistical Analysis

All experiments in the study were performed at least three times, and the results were reported as mean ± SD. The statistical significance among different treatments in each individual experiment was compared by ANOVA using SPSS 13.0 statistical-analysis system. A value of *p* < 0.05 was considered to be significantly different.

## 4. Conclusions

This study presents encapsulation mechanism of oxyresveratrol by β-CD and HP-β-CD. Combined with NMR analysis and molecular docking simulation, the most possible mode of interactions between oxyresveratrol and CDs were shown. Based on the results of this research, oxyresveratrol formed 1:1 complexes with both β-CD and HP-β-CD. However, *K_F_* value of HP-β-CD was significantly higher than that of β-CD. The *K_F_* values exhibited a strong dependence on temperature, which decreased as the system temperature increasing. The thermodynamic study demonstrated that the complexation processes between oxyresveratrol and CDs were spontaneous and exothermic. A low temperature is favorable for the inclusion process and for improving the stability of the inclusion complex. In addition, the encapsulation constants between CDs and oxyresveratrol have a sharp decrease in the pH region where the hydroxyl groups of oxyresveratrol deprotonation. Moreover, according to molecular docking combined with ^1^H-NMR analysis, oxyresveratrol-β-CD and oxyresveratrol-HP-β-CD inclusion complexes could be formed by penetrating aromatic rings of guest molecule into the cavity from the wide rim of cavity partly, while the protons of oxyresveratrol in HP-β-CD complex presented stronger H-bond interaction than β-CD.

## Figures and Tables

**Table 1:** Thermodynamic parameters for oxyresveratrol-β-CD and oxyresveratrol-HP-β-CD inclusion complexes from phase solubility diagrams at different temperatures.

	*ΔG*^0^ (KJ/mol)		*ΔS*^0^ (J/mol·K)		*ΔH*^0^ (KJ/mol)	
T (°C)	β-CD	HP-β-CD	β-CD	HP-β-CD	β-CD	HP-β-CD
20	−18.70 ± 0.10	−25.98 ± 0.27	−85.25 ± 0.37	−192.07 ± 0.94	−47.60 ± 2.38	−91.53 ± 7.86
30	−17.30 ± 0.28	−22.89 ± 1.20	−90.02 ± 0.97	−202.63 ± 4.09		
40	−15.85 ± 0.61	−20.17 ± 0.78	−94.99 ± 2.10	−211.91 ± 3.49		
50	−13.97 ± 0.91	−16.90 ± 0.51	−101.38 ± 3.04	−223.07 ± 3.37		
60	−13.01 ± 0.83	−15.23 ± 0.21	−104.37 ± 2.82	−228.78 ± 0.73		

Results are represented as mean ± SD (*n* = 3).

**Table 2:** Variation of the ^1^H chemical shift (δ, ppm) of oxyresveratrol and β-CD as well as HP-β-CD protons in free and complex states.

Substance	Proton	Free (δ, ppm)	Oxyresveratrol-β-CD (δ, ppm)	Δδ ^a^ (ppm)	Oxyresveratrol-HP-β-CD (δ, ppm)	Δδ ^a^ (ppm)
Oxyresveratrol	H-α	7.006	6.893	−0.113	6.870	−0.136
	H-β	7.361	7.366	0.005	7.325	−0.036
	H-1	-	-	-	-	-
	H-2	6.690	6.607	−0.083	6.544	−0.146
	H-3	-	-	-	-	-
	H-4	6.275	6.415	0.140	6.319	0.044
	H-5	-	-	-	-	-
	H-6	6.690	6.607	−0.083	6.544	−0.146
	H-1’	-	-	-	-	-
	H-2’	-	-	-	-	-
	H-3’	6.486	6.551	0.065	6.492	0.006
	H-4’	-	-	-	-	-
	H-5’	6.547	6.548	0.001	6.482	−0.065
	H-6’	7.540	7.469	−0.071	7.424	−0.116
β-CD	H-3	4.052	3.997	−0.055	-	-
	H-5	3.951	3.906	−0.045	-	-
HP-β-CD	H-3	4.035	-	-	4.014	−0.021
	H-5	3.750	-	-	3.746	−0.004

^a^ The chemical shift change(Δδ) is defined as the difference between δ_complex_ and δ_free_, Δδ = δ_complex_ − δ_free_.

**Table 3:** The lowest energy values for the complex of oxyresveratrol and different CDs based on Autodock results.

	Intermolecular Energy (kcal/mol)	Internal Energy (kcal/mol)	Torsional Energy (kcal/mol)	Unbound Energy (kcal/mol)	Binding Energy ^a^ (kcal/mol)
β-CD	−8.57	−0.16	1.79	−0.16	−6.78
HP-β-CD	−9.27	−0.26	1.79	−0.26	−7.48

^a^ Binding Energy = Intermolecular Energy + Internal Energy + Torsional Energy − Unbound Energy.
